# Autophagy is influenced by vitamin D_3_ level in people with HIV-1

**DOI:** 10.1186/s13062-025-00660-9

**Published:** 2025-06-13

**Authors:** Rita Casetti, Fabiola Ciccosanti, Harpreet Kaur Lamsira, Carmela Pinnetti, Valentina Mazzotta, Serena Ciolfi, Alessandra Sacchi, Alessandra Amendola, Giuseppe Ippolito, Mauro Piacentini, Roberta Nardacci

**Affiliations:** 1 Department of Epidemiology, Preclinical Research and Advanced Diagnostics, National Institute for Infectious Diseases ’Lazzaro Spallanzani’ - IRCCS, Rome, 00149 Italy; 2 Departmental Faculty of Medicine, Saint Camillus International University of Health Sciences, Rome, 00131 Italy; 3 Clinical and Research Department, National Institute for Infectious Diseases ‘Lazzaro Spallanzani’-IRCCS, Rome, 00149 Italy; 4 Department of Science, University of Roma Tre, Rome, 00146 Italy; 5 Laboratory of Virology and Biosafety Laboratories, National Institute for Infectious Diseases ‘Lazzaro Spallanzani’- IRCCS, Rome, 00149 Italy; 6 Department of Biology, University of Rome ‘Tor Vergata’, Rome, 00133 Italy

**Keywords:** Autophagy, AMBRA1, BECN1, ATG5, Vitamin D₃, HIV-1, ART

## Abstract

**Background:**

Autophagy is the primary catabolic process responsible for degrading intracellular components and potentially harmful cytosolic entities by delivering them to lysosomes. Notably, this mechanism is crucial for controlling intracellular pathogens, with significant implications for both innate and adaptive immunity. In the context of HIV-1 infection, emerging evidence suggests that autophagy contributes to immune responses against the virus. Various compounds can modulate autophagy, among which vitamin D₃ is particularly effective due to its ability to prevent inflammation and slow HIV-1 disease progression. Indeed, vitamin D₃ contributes to regulating both innate and adaptive immunity, thereby modulating antiviral and antibacterial inflammatory responses. Importantly, vitamin D₃ deficiency is highly prevalent among people with HIV (PWH) and has been associated with an increased risk of severe disease progression.

**Results:**

In this study, we investigated the relationship between serum vitamin D₃ levels and the expression of autophagy markers in peripheral blood mononuclear cells from different categories of PWH: PWH under antiretroviral therapy (ART) with either normal vitamin D₃ levels or hypovitaminosis, and treatment-naïve PWH with either normal vitamin D₃ levels or hypovitaminosis. Our results show that low vitamin D₃ blood levels is associated with lower expression of the main factors involved in the autophagy mechanism, particularly in treatment-naïve PWH.

**Conclusions:**

Our findings suggest that normal blood level of vitamin D₃ may play a crucial role in promoting autophagy in PWH. The observed differences in autophagy-related protein expression between ART-treated and untreated individuals underscore the intricate relationship between vitamin D₃ levels, ART exposure, and autophagic regulation. This is a preliminary exploration of the effects of vitamin D₃ on autophagy in PWH. Further studies are needed to deepen and explore the interplay between vitamin D₃ and autophagy in greater depth. A better understanding of these mechanisms could help to develop novel therapeutic strategies aimed at mitigating immune depletion and chronic inflammation, ultimately improving clinical outcomes for individuals living with HIV.

**Supplementary Information:**

The online version contains supplementary material available at 10.1186/s13062-025-00660-9.

## Background

Autophagy is a physiological process that facilitates the degradation of cytoplasmic components and microbial pathogens, including viruses, through their delivery to lysosomes [1]. This mechanism, also defined as xenophagy, plays a crucial role in host immune defense by eliminating and degrading invading pathogens. However, many pathogens have evolved sophisticated strategies to evade or suppress the immune-supportive functions of xenophagy and even exploit autophagy-related proteins to their advantage [2, 3].

Autophagy functions as a double-edged sword in HIV-1 infection, influencing viral replication, immune response modulation, and the fate of infected cells. Research suggests that during the early stages of HIV infection, the virus manipulates autophagy pathways to render host cells more susceptible to infection. By contrast, dysregulation of autophagy in the later stages of the viral replication cycle may lead to the autophagic cell death of CD4 + T cells or contribute to the establishment of a state of a viral latency in the host cell, both of which are key factors in disease progression and viral persistence [4–6].

While autophagy exhibits diverse functions in HIV-1 infection, emerging evidence suggests that its stimulation may enhance immune responses against the virus [7, 8]. Autophagy has been shown to play a role in controlling HIV-1 infection among long-term non-progressors (LTNPs), individuals who maintain stable health without antiretroviral therapy, and elite controllers (ECs), individuals who maintain stable health and spontaneously control the viral replication in the absence of therapy. Notably, peripheral blood mononuclear cells (PBMCs) from LTNPs and ECs exhibit higher levels of autophagic activity compared to normal progressors, suggesting a potential role for autophagy in viral control [9].

Autophagy, along with programmed cell death, plays a central role in viral persistence and immune dysfunction. Furthermore, challenges related to viral reservoirs and drug resistance continue to impede the effective management of HIV-1 infection [10].

This study aims to examine the relationship between serum vitamin D₃ levels and the expression of the autophagy markers, namely AMBRA1, BECN1, ATG5 and LC3, exploring the potential of vitamin D₃ [11, 12] as a strategy to counteract HIV-1 infection.

Vitamin D₃ is widely recognized for its numerous extra-skeletal functions, including its roles in cell differentiation, proliferation, antimicrobial activity, antioxidant defence, and immunomodulation across various tissues and cell types [13]. It plays a crucial role in regulating both innate and adaptive immunity, thereby influencing antiviral and antibacterial immune responses [14–16].

The antimicrobial effects of vitamin D are well documented, and several studies have linked vitamin D deficiency to an increased risk of severe HIV-1 infection [17, 18]. Notably, vitamin D deficiency is prevalent among individuals living with HIV due to multiple factors, including chronic inflammation, HIV-related comorbidities, and the impact of antiretroviral drugs [18–20].

Indeed, low vitamin D levels have been associated with increased HIV-1-related mortality, accelerated disease progression, osteopenia/osteoporosis, and virological failure following the initiation of ART [4].

Despite all this scientific evidence, the current literature remains inconclusive regarding the effectiveness of vitamin D₃ supplementation in improving HIV-related outcomes.

In this study, we investigated the relationship between serum vitamin D₃ levels and the expression of autophagy markers in blood cells from distinct categories of PWH.

Modulating autophagy in PWH may represent a promising therapeutic avenue for combating the virus and potentially achieving viral eradication. Despite significant advancements in cART, HIV-1 infection remains a major global health challenge, with approximately 38.4 million people currently living with the virus. Although ART has remarkably succeeded in extending the lifespan and improving the quality of life for PWH, several challenges remain, including viral persistence, drug resistance, and long-term adverse effects of antiretroviral drugs [21–23].

A deeper understanding of the molecular mechanisms governing autophagy in the context of HIV-1 infection is crucial for deciphering host-virus interactions. Such insights could pave the way for novel clinical interventions aimed at viral eradication and immune system restoration.

## Methods

### Patients enrolled

Venous blood was collected from adult human subjects receiving care at the National Institute for Infectious Diseases (INMI) “Lazzaro Spallanzani”. All participants provided written informed consent to be enrolled in the study (Ethics Committee approval n° 40/2024).

In this paper we analyzed four categories of patients: PWH naïve for cART with normal level of 25(OH)D (level as > 75 nmol/L) (20 patients), PWH naïve for cART with hypovitaminosis D (level as < 50 nmol/L) (20 patients), PWH cART treated with normal level of 25(OH)D (level as > 75 nmol/L) (20 patients) and PWH cART treated with hypovitaminosis D (level as < 50 nmol/L) (20 patients).

Patients naïve for ART treatment showed a median viral load (plasma HIV-1 RNA) of 4.5 ± 0.6 log₁₀ copies/mL HIV-RNA and CD4⁺ T cell counts below 500 cells/µL, PWH receiving cART showed values of viral load of < 50 copies/mL HIV-RNA and CD4⁺ T cell counts above 500 cells/µL; PWH were of both sexes, mean age 39 years. Circulating 25-hydroxyvitamin D [25(OH)D] was quantified in the different patient’s categories.

Plasma HIV-1 RNA levels were measured with Aptima HIV-1 Quant Dx assay (Hologic.com), according to the manufacturer’s instructions.

PBMCs were isolated by Ficoll/Hypaque (GE Healthcare, 17-1440-02) centrifugation of heparinized whole blood and partly used immediately for immonocytochemical analysis, and the remaining part frozen at -80 °C for subsequent western blot experiments.

### Immunocytochemistry

PBMCs were isolated and fixed in 4% freshly depolymerized paraformaldehyde (Sigma-Aldrich, P-6148) in PBS (Sigma-Aldrich, P-4417) pH 7.2.

Immunocytochemistry was performed as previously reported [24] on fixed PBMCs. The primary antibodies utilized were: rabbit polyclonal anti-beclin 1 (BECN1) (1:500, Santa Cruz Biotechnology, sc-11427), rabbit polyclonal anti-autophagy and beclin 1 regulator 1 (AMBRA1) (1:100, ProSci 4557), rabbit polyclonal anti-autophagy related 5 (APG5) (1: 200, Santa Cruz Biotechnology, sc-33210), rabbit polyclonal anti-microtubule associated protein 1 light chain 3 beta (LC3B) (1:2000, Cell Signaling, 3868).

For each staining, the percentage of positive PBMCs/total PBMCs was quantified. Three independent observers evaluated the number of positive cells by using a light microscope without the knowledge of clinical diagnosis. A minimum of 500 PBMCs/patients were analyzed.

### Immunofluorescence

For immunofluorescence experiments, PBMCs were incubated with mouse monoclonal anti-LC3B (1:50, Cosmo Bio, CTB-LC3-1-50). Specimens were thoroughly rinsed with PBS, then incubated for 1 h at room temperature with 1:400 Alexa 488 conjugated goat anti-mouse IgG (H + L) (Invitrogen, Life Technologies, A-11029). Controls were performed by omitting the primary antibody. Slides were observed and photographed in a Leica TCS SP2 confocal microscope (Leica Microsystems GmgH, Ernst-Leitz-trasse 17–37 35578 Wetzlar Germany).

The percentage of cells containing LC3-positive dots was evaluated. A minimum of 30 cells/patients were observed. Cell counting was done by 3 independent individuals; data are presented as mean *±* S.D.

### Western blot analysis

Total proteins were extracted from PBMCs, from individual patients, by using the Nuclear Extract Kit (Active Motif, Carlsbad, CA, USA, 40010) and resolved by electrophoresis through 4–12% Bis-Tris Plus gel (Invitrogen, Rockford, IL, USA, NW04120BOX) and electroblotted onto nitrocellulose (Bio-RadLaboratories, Hercules, CA, USA, 170–4158) or PVDF (Millipore, IPVH20200) membranes. Blots were incubated with 50 mg/mL non-fat dry milk in buffered saline plus 0,1% Tween-20 (TBST) and then in primary antibodies diluted in 50 mg/mL non-fat dry milk in TBST, overnight, at 4 ◦C. Primary antibodies were: rabbit polyclonal anti-AMBRA1 (1:2000, Novusbio, 6190002), rabbit polyclonal anti-BECN1 (1:500, Santa Cruz Biotechnology, sc-11427), rabbit polyclonal anti-APG5 (1: 200, Santa Cruz Biotechnology, sc-33210), rabbit polyclonal anti-LC3B (1:2000, Cell Signaling, 3868) anti-GAPDH (1:60000, Calbiochem, CM1001). Detection was achieved using the specific horseradish peroxidase-conjugate secondary antibody (anti mouse IgG 1:5000, Santa Cruz Biotechnology, sc-2005; or anti rabbit IgG 1:5000, Santa Cruz Biotechnology, sc-2004) and visualized with Clarity Western ECL (Biorad, 170–5060). Mouse anti-GAPDH antibody was used to monitor equal protein loading.

Western blot images were analyzed densitometrically using ChemiDoc Touch Imaging System (Bio-RadLaboratories, Hercules, CA, USA) and processed with ImageJ software to quantify bands intensity.

### Statistical analysis

To determine statistical significance, the Student *t* test and Pearson coefficient correlation were used. Statistical significance was set at *p* < 0.05.

## Results

### Immunocytochemical analysis of autophagic factor expression in PBMCs from PWH

We evaluated the expression of BECN1, AMBRA1 and ATG5 and by immunohistochemistry in PBMCs isolated from PWH stratified based on cART treatment and Vitamin D blood levels.

**Fig. 1:**
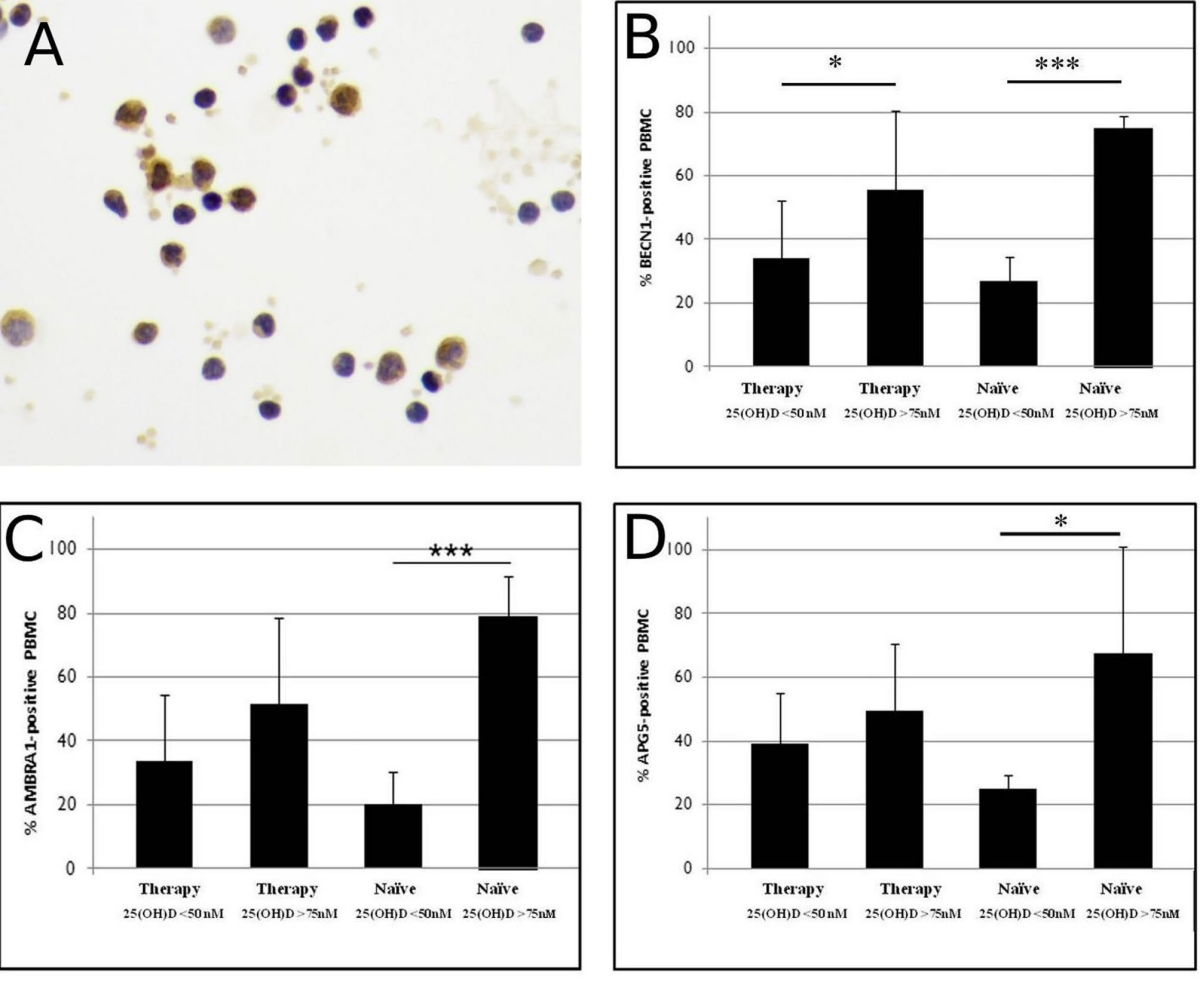
Expression of autophagy-related factors in PBMC from PWH on therapy and treatment-naїve individuals with hypovitaminosis or normal vitamin D_3_ blood level (**A**). Immunocytochemical localization of BECN1 (**B**), AMBRA1 (**C**) and APG5 (**D**) is shown. The graph shows the mean values of 3 independent experiments performed on PBMCs from PWH, both on therapy and treatment-naїve, with either hypovitaminosis or normal vitamin D_3_ blood concentration. Data are reported as mean values ± S.D. * *p* < 0.05, ** *p* < 0.01, *** *p* < 0.001. Original magnification: A, 40X

Figure 1A shows a representative image of AMBRA1 immunolabeling. The quantification of labelled PBMCs was performed using antibodies against BECN1 (Fig. 1B), AMBRA1 (Fig. 1C), and APG5 (also known as ATG5) (Fig. 1D).

In PWH with normal levels of vitamin D₃ (> 75 nmol/l), high percentage of labelled PBMCs for all the three main autophagy markers was observed, independently from cART assumption. Those with hypovitaminosis, instead, showed lower percentage of labelled cells. Notably, this difference was statistically significant by comparing the percentage of positive cells in naïve patients with vitamin D₃ levels > 75 nmol/L with that of PWH with vitamin D3 < 50 nmol/L. In treated patients a higher level of BECN1 was observed in individuals with normal vitamin D₃ than those with hypovitaminosis. A similar trend (not statistically significant) was found for AMBRA1 and ATG5.

### Quantification of autophagic proteins in PBMCs from PWH by Western blot analysis

To validate the immunocytochemistry findings, we assessed the expression levels of BECN1, AMBRA1, APG5, and LC3-II in PBMCs from patients of each categories using Western blot (WB) analysis.

**Fig. 2:**
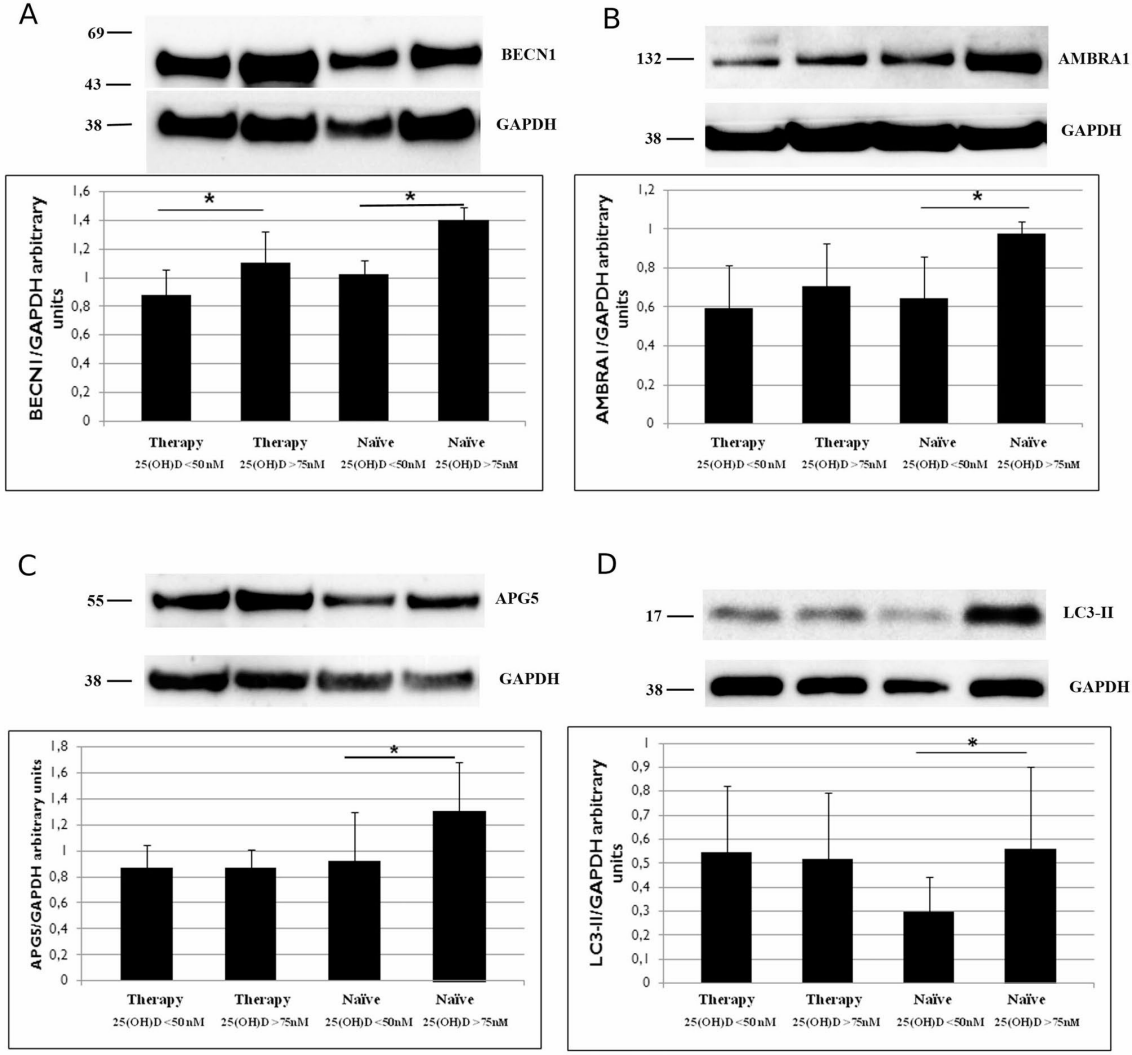
Western Blotting analysis of BECN1 (**A**), AMBRA1 (**B**) APG5 (**C**) and LC3 II (**D**) levels on PBMCs from PWH on therapy and treatment-naїve individuals with hypovitaminosis or normal vitamin D_3_ blood level. In the graph are shown the mean values from 3 independent experiments. Data are reported as arbitrary units ± S.D. * *p* < 0.05

Densitometric analysis revealed a significantly higher expression of BECN1 (Fig. 2A) in both treated and treatment-naïve patients with normal vitamin D₃ levels compared to those with hypovitaminosis (*p* < 0.05).

Regarding AMBRA1 expression (Fig. 2B), densitometric analysis showed a higher level in treatment-naїve patients with a normal concentration of vitamin D_3_ compared to patients who showed hypovitaminosis (*p* < 0.05). Differently, no difference was revealed comparing treated patients.

Similarly to AMBRA1, APG5 (Fig. 2C) and for LC3-II expression (Fig. 2D) did not differ between treated patients with normal vitamin D₃ levels and those with hypovitaminosis D₃. However, a significantly higher expression of both markers was observed in treatment-naïve patients with normal vitamin D₃ levels compared to those with hypovitaminosis D₃ (*p* < 0.05).

### Immunofluorescence analysis of the autophagic marker LC3B in PBMCs from PWH

**Fig. 3:**
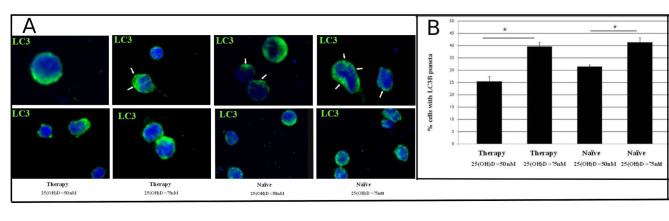
Confocal microscopy immunolocalization of the autophagosome marker LC3B (green) on PBMC from PWH on therapy and treatment-naїve individuals with hypovitaminosis or normal vitamin D_3_ blood level. The white arrows indicate the LC3B puncta on PBMC (**A**). Quantitative analysis of the percentage of cells showing LC3B dots in the cell cytoplasm (**B**). In the graph are shown the percentage of labelled PBMCs from PWH on therapy and treatment-naїve with hypovitaminosis or normal vitamin D_3_ blood concentration. Data are reported as mean values ± S.D. * *p* < 0.05. Original magnification: A, 63 X

To further confirm the lower autophagy level in PWH with hypovitaminosis, the autophagy marker LC3B was analyzed by immunofluorescence across all patient categories (Fig. 3A). The percentage of PBMCs containing LC3 puncta was quantified, revealing a significantly higher proportion of LC3 puncta-positive cells in both treated and treatment-naïve patients with normal vitamin D₃ levels compared to those with hypovitaminosis (Fig. 3B). These data suggest the active autophagic processes within cells from patients with normal vitamin D₃ levels.

## Discussion

HIV-1 persists in infected patients despite antiretroviral therapy due to the presence of latent viral reservoirs, primarily within resting CD4 + T cells and other immune cell populations (Alum et al., 2024). These reservoirs pose a significant barrier to achieving an HIV-1 eradication or cure. Recent research has explored the potential of leveraging cellular processes such as autophagy to target and eliminate these reservoirs [5, 7–9]. Indeed, this fundamental cellular degradation pathway has emerged as a promising mechanism for reducing or eradicating latent HIV-1 reservoirs [25].

In this study, we investigate the relationship between serum vitamin D₃ levels and the expression of autophagy markers, evaluating the potential of vitamin D₃, a well-established autophagy activator [11], to sustain the autophagy as a strategy to counteract HIV-1 infection.

Vitamin D₃ levels in PWH vary widely. Observational studies report a prevalence of vitamin D deficiency in this population ranging from 24 to 81% [19]. Several factors contribute to this deficiency, including non-virus-related risk factors such as gender, age, and sun exposure. Additionally, certain antiretroviral drugs, particularly protease inhibitors (PIs) and non-nucleoside reverse transcriptase inhibitors (NNRTIs), can disrupt vitamin D metabolism [26].

Building on these observations, we analyzed the expression of key autophagic markers in PBMCs from PWH with different serum vitamin D₃ concentrations.

Our findings seem to demonstrate a strict association between normal blood level of vitamin D₃ and the expression of key autophagy-related proteins in PBMCs from PWH. Immunocytochemical analysis of autophagic factors revealed a higher percentage of labelled PBMCs in both cART-treated and treatment-naïve PWH with normal vitamin D₃ blood levels compared to those with hypovitaminosis. Notably, the percentage of PBMCs positive for AMBRA1, APG5, and BECN1 in treatment-naïve PWH with vitamin D₃ levels > 75 nmol/L was comparable to that observed in PBMCs from long-term non-progressors (LTNPs) and elite controllers (ECs) [9]. These last individuals, who represent a small subset of PWH (5–15%), remain clinically stable for years without antiretroviral therapy and are classified as non-progressors [27].

Remarkably, the proportion of autophagy marker-positive cells in these treatment-naïve PWH with sufficient vitamin D₃ levels reached nearly 80%, a percentage previously reported for the same autophagic factors in LTNPs. This finding is particularly significant, as it suggests that in treatment-naïve PWH with normal vitamin D₃ levels, the autophagic activity remain preserved similarly to that observed in LTNPs [9]. This similarity suggests that adequate dose of vitamin D₃ may play a key role in sustain autophagic activity, potentially contributing to better control of the HIV-1 replication.

According to our results, a previously published paper demonstrated a significant downregulation of transcript levels of LC3, ATG5 and BECN1 mRNA in HIV-positive subjects with 25(OH)D3 deficiency compared with HIV-positive subjects with adequate 25(OH)D3 levels [17].

These findings align with other previous studies indicating that vitamin D₃ can induce autophagy in human macrophages and can be involved in the inhibition of HIV-1 replication. For instance, Campbell and Spector (2011) demonstrated that hormonally active vitamin D₃ triggers autophagy in macrophages, thereby reducing HIV-1 infection. Furthermore, another study by Campbell and Spector [28] reported that vitamin D inhibits HIV-1 and Mycobacterium tuberculosis infections in macrophages through the induction of autophagy.

The results obtained in this study through immunocytochemistry and confirmed by western blot analysis and confocal microscope analysis, indicate a close relationship between the dose of circulating vitamin D_3_ and the levels of autophagy factors in PBMCs of PWH.

BECN1, a core component of the autophagy initiation complex, was significantly more expressed in both cART-treated and treatment-naïve patients with normal vitamin D₃ levels. This suggests that sufficient vitamin D₃ levels may help sustain autophagy initiation. The expression of AMBRA1 followed a similar pattern, with higher levels observed in treated persons with normal vitamin D₃ levels compared to those with hypovitaminosis. More strikingly, treatment-naïve PWH with normal vitamin D₃ levels exhibited significantly higher AMBRA1 expression. This suggests that vitamin D₃ may exert a direct regulatory effect on autophagy activation, particularly in the absence of cART, which is known to influence cellular metabolic pathways and immune responses.

Interestingly, while APG5 expression did not differ significantly between ART-treated PWH with normal vitamin D₃ levels and those with hypovitaminosis, treatment-naïve PWH with normal vitamin D₃ levels exhibited significantly higher APG5 expression. This finding suggests that the impact of vitamin D₃ on autophagy may be more pronounced in untreated PWH, possibly due to the absence of cART that can induce metabolic alterations that could interfere with autophagy regulation.

We analyzed the expression of LC3, a member of the autophagy-related protein 8 (ATG8) family, which plays a central role in canonical autophagy by regulating autophagosome formation, cargo recognition, and fusion with lysosomes. The conversion of LC3 from its cytosolic, unconjugated form (LC3-I) to the phosphatidylethanolamine-conjugated form (LC3-II) is a key event in autophagosome formation.

Our data showed that LC3-II expression in cART-treated PWH did not differ significantly between individuals with normal serum vitamin D₃ levels and those with hypovitaminosis D. In contrast, treatment-naïve PWH with normal vitamin D₃ levels exhibited significantly higher LC3-II expression than those with vitamin D₃ deficiency, suggesting that vitamin D₃ may modulate autophagy in these patients.

It is important to note that the interpretation of autophagy markers such as LC3-II requires complementary assays to assess autophagic flux to permit a correct interpretation of the results. Autophagic activity encompasses not only the synthesis and lipidation of LC3, but more critically, the entire dynamic process, including lysosomal fusion and degradation of autophagic cargo. An accumulation of LC3-II may also reflect impaired autophagosome clearance due to defects in lysosomal fusion or lysosomal dysfunction. This represents a limitation of our observational study.

Further investigations are needed, such as ex vivo pharmacological modulation of autophagy in PBMCs to better characterize autophagic flux and confirm our findings.

We analysed LC3B expression through immunofluorescence and provided insights into the autophagic activity in PBMCs from PWH. Our results demonstrate a significantly higher percentage of PBMCs exhibiting LC3 puncta in both treated and treatment-naïve PWH with normal vitamin D₃ levels compared to those with hypovitaminosis, suggesting that vitamin D₃ may play a role in modulating autophagic activity in the context of HIV-1 infection. The higher proportion of LC3 puncta-positive cells observed in patients with normal vitamin D₃ levels is consistent with the notion that sufficient vitamin D₃ availability may support autophagic processes that contribute to better immune control of HIV-1 infection.

These findings align with previous research indicating that vitamin D₃ can stimulate autophagy, potentially enhancing the immune response against HIV-1 [8, 11, 17].

A recently published paper demonstrated the beneficial effects of Vitamin D in reducing HIV infection. Vitamin D appears to restrict viral replication by modulating the transcription of antiviral and metabolic genes [29].

A limit of the analysis of LC3B expression by immunofluorescence, is that the higher percentage of PBMCs displaying LC3 puncta in both treated and treatment-naïve PWH with normal vitamin D₃ levels may reflect a downstream blockade of autophagic flux. Therefore, as previously noted, further in-depth studies are required to rule out this possibility. Ex vivo and in vitro experiments, involving the pharmacological modulation of autophagy, will be essential to clarify this molecular mechanism and to further investigate the potential role of vitamin D₃ in reducing HIV-1 replication.

However, our data add a further piece to the picture of xenophagy: namely that in PLWH autophagic activity is maintained in the presence of normal doses of vitamin D_3_ in the serum, providing new insights into potential mechanisms of autophagy to counteract the HIV-1 pathogenesis.

A particularly intriguing mechanistic pathway potentially linking vitamin D₃ to enhanced autophagy involves the transcription factor EB (TFEB), a master regulator of lysosomal biogenesis and autophagy. TFEB is regulated by the mechanistic target of rapamycin (mTOR), which phosphorylates TFEB to retain it in the cytoplasm. Conversely, the phosphatase PPP3/calcineurin dephosphorylates TFEB, allowing its translocation into the nucleus, where it activates the transcription of numerous autophagy-related genes [30]. Kumar et al. (2020) uncovered the role of mAtg8s as regulators of the lysosomal system acting upstream of TFEB. They identified a key regulatory node involving mammalian Atg8 proteins (mAtg8s), in particular the GABARAP subfamily and the immunity-related GTPase IRGM that were shown to directly interact with TFEB, promoting its nuclear translocation and transcriptional activity. Autophagy immune functions include direct elimination of intracellular microbes and control of inflammation. IRGM is required for the full activation of TFEB in response to Mycobacterium tuberculosis infection in macrophages. Notably, the HIV-1 accessory protein Nef can modulate TFEB function in an IRGM-dependent manner [31].

Although our study did not directly assess TFEB activity, the observed upregulation of the key autophagy markers BECN1, AMBRA1, ATG5, and LC3 in PWH with sufficient vitamin D₃ levels is consistent with enhanced TFEB-mediated transcription. These findings suggest a model in which vitamin D₃ may promotes autophagy through upstream modulation of the mTOR–TFEB axis, potentially by influencing the activity of GABARAPs and IRGM. Targeting this regulatory network may represent a promising therapeutic avenue to restore autophagy and strengthen immune control in chronic viral infections.

Further studies are warranted to investigate TFEB localization and phosphorylation status, as well as the expression and activity of IRGM and GABARAP proteins, in the context of vitamin D₃ supplementation during HIV-1 infection.

Overall, the findings of this study suggest a promising avenue for clinical translation, supporting the potential role of vitamin D₃ supplementation as an adjunctive therapy to cART in the management of HIV-1 infection.

Vitamin D₃ supplementation could represent a possible adjunctive therapy for treating HIV-1, particularly in PWH with low vitamin D₃ levels, by restoring or enhancing autophagic activity and improving immune function. Furthermore, maintaining adequate vitamin D₃ levels may be especially important for PWH, who face an increased risk of osteopenia, malignancies, and cardiovascular disease [16, 32–34].

Well-controlled randomized clinical trials remain essential to evaluate the efficacy of vitamin D₃ supplementation as an adjunctive therapy in PWH [34–36]. Additionally, further research is needed to elucidate the precise mechanisms through which vitamin D₃ modulates autophagy in the context of HIV-1 infection and to explore its potential as a therapeutic strategy for enhancing immune function and reducing viral reservoirs.

### Limitations of the study

The main limit of our study is its observational nature. This is a preliminary exploration of the effects of vitamin D₃ on autophagy in PWH. Further studies are required to achieve a more comprehensive understanding of autophagic flux and to better evaluate the potential of vitamin D₃ as a therapeutic strategy to counteract HIV-1 infection.

## Conclusions

All together, these data suggest that the serum levels of vitamin D₃ may influence the autophagy activity in PBMCs of PWH. The observed differences in autophagy-related protein expression between cART-treated and untreated PWH highlight the complex interplay between vitamin D₃ levels, cART, and autophagic regulation, providing novel insights into the potential mechanisms underlying HIV-1 pathogenesis.

A deeper understanding of these mechanisms could contribute to the development of innovative therapeutic strategies aimed at mitigating immune depletion and chronic inflammation, ultimately improving clinical outcomes for PWH.

## Electronic supplementary material

Below is the link to the electronic supplementary material.

Supplementary Material 1

Supplementary Material 2

Supplementary Material 3

Supplementary Material 4

## Data Availability

No datasets were generated or analysed during the current study.
