# Supplementary figures and images for: Autophagy is influenced by vitamin D3 level in people with HIV-1
Source: Biol Direct. 2025 Jun 13;20:69. doi: 10.1186/s13062-025-00660-9 (PMC12164164; doi:10.1186/s13062-025-00660-9)

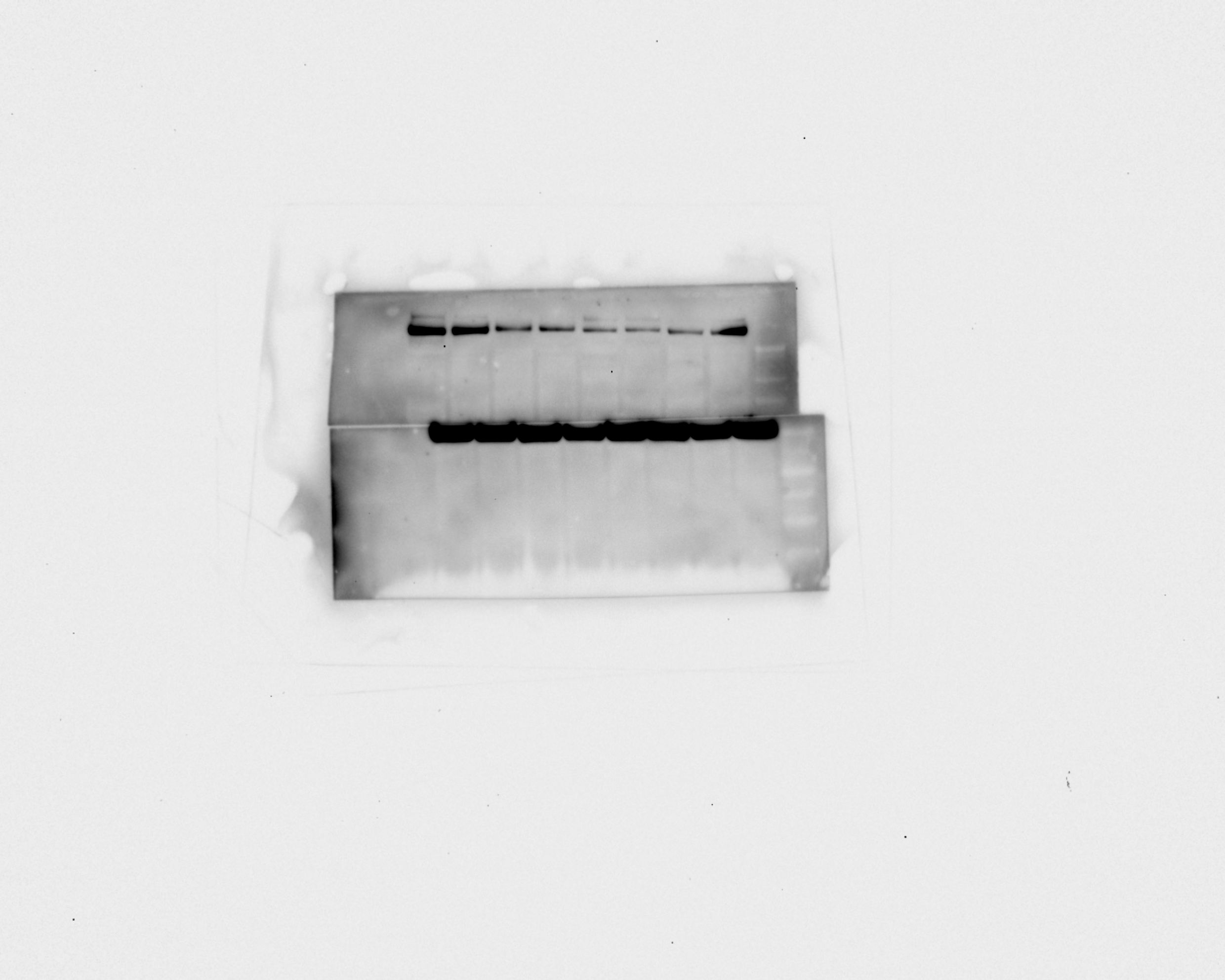

Supplement: Supplementary file 1 — Supplementary Material 1 [file 13062_2025_660_MOESM1_ESM.jpg]

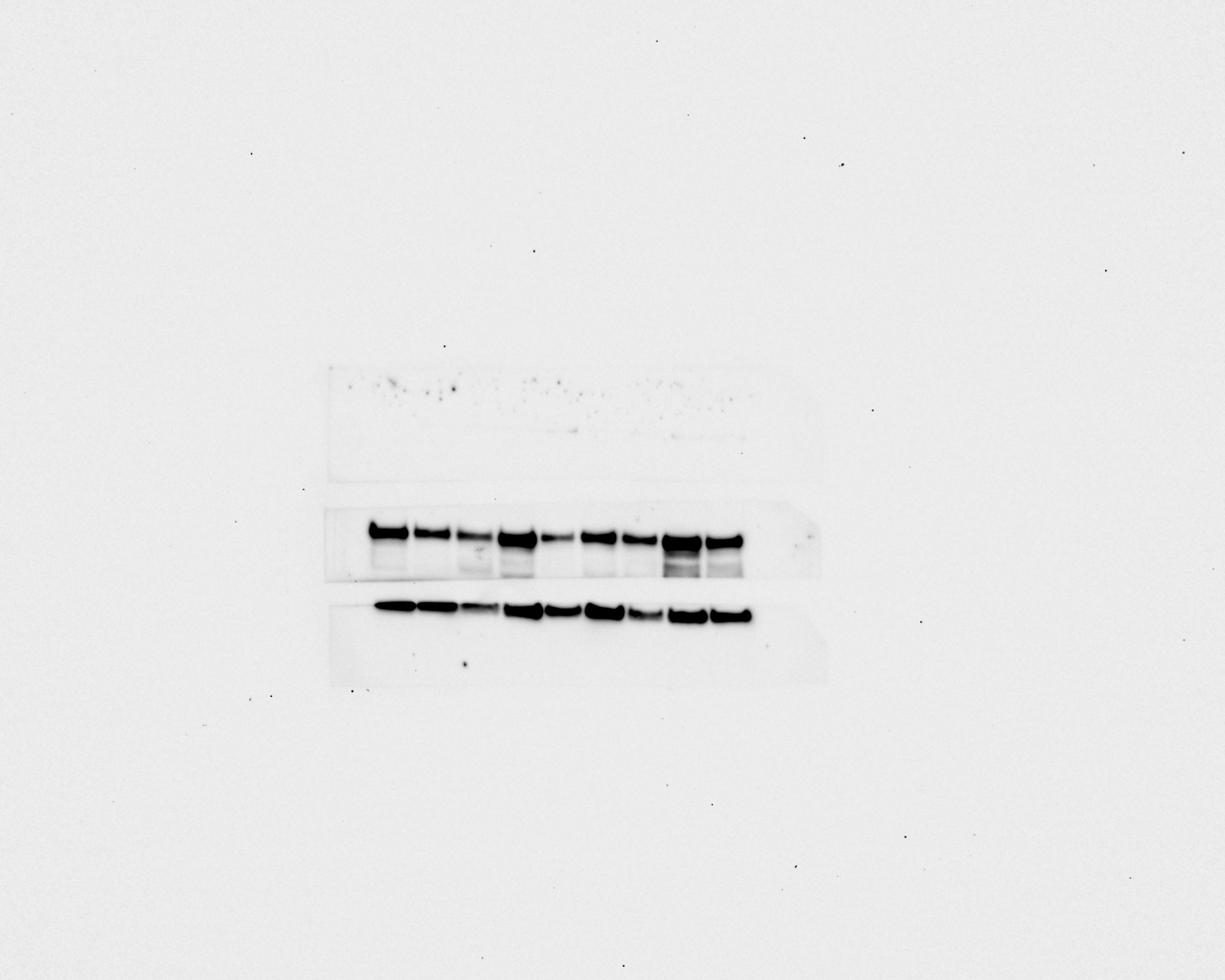

Supplement: Supplementary file 2 — Supplementary Material 2 [file 13062_2025_660_MOESM2_ESM.jpg]

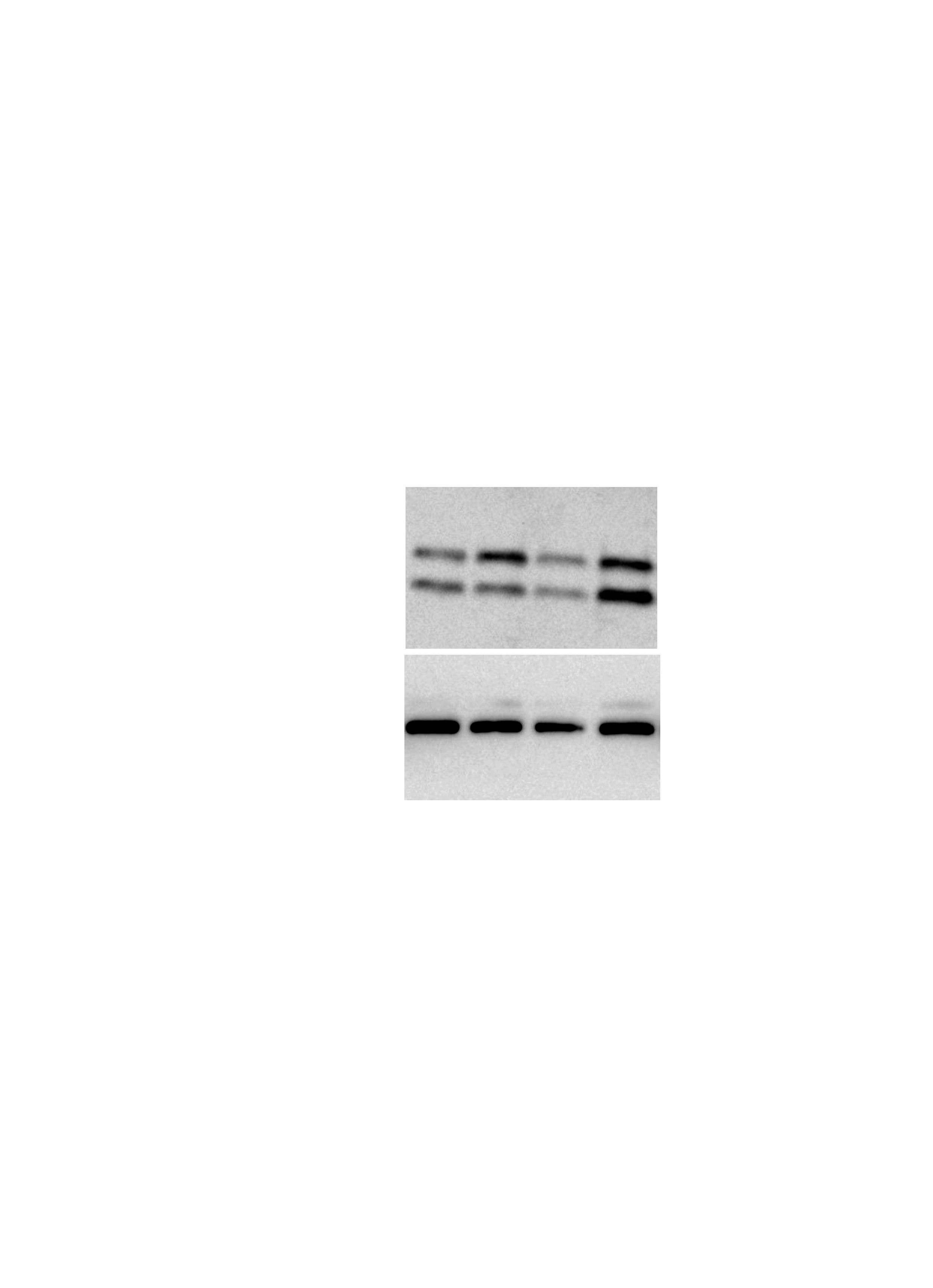

Supplement: Supplementary file 3 — Supplementary Material 3 [file 13062_2025_660_MOESM3_ESM.jpg]

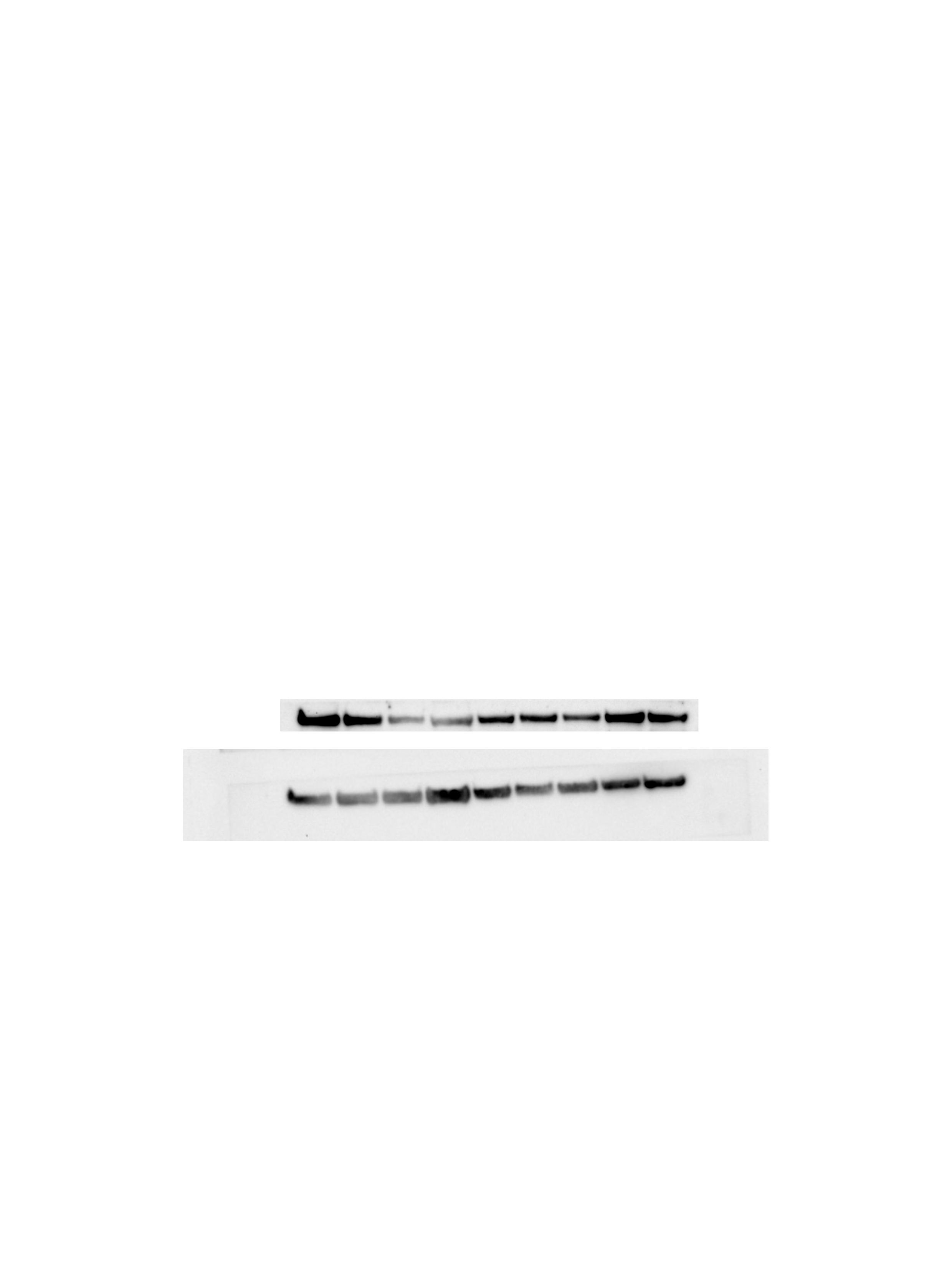

Supplement: Supplementary file 4 — Supplementary Material 4 [file 13062_2025_660_MOESM4_ESM.jpg]
